# Beyond birth rates: a theoretical framework for women’s reproductive autonomy and quality childbearing

**DOI:** 10.3389/fpubh.2026.1734790

**Published:** 2026-04-02

**Authors:** Zhaohui Su, Xue Yang, Dean McDonnell, Junaid Ahmad, Barry L. Bentley, Ali Cheshmehzangi, Sabina Šegalo, Jing-Bao Nie, Claudimar Pereira da Veiga, Yu-Tao Xiang

**Affiliations:** 1 Key Laboratory of Environmental Medicine Engineering, Ministry of Education, School of Public Health, Southeast University, Nanjing, China; 2 School of Public Health, Southeast University, Nanjing, China; 3 Department of Humanities, South East Technological University, Carlow, Ireland; 4 School of Public Affairs, Zhejiang University, Hangzhou, China; 5 Bioengineering Research Group, Cardiff School of Technologies, Cardiff Metropolitan University, Cardiff, United Kingdom; 6 Collaboration for the Advancement of Sustainable Medical Innovation, University College London, London, United Kingdom; 7 School of Architecture, Design and Planning, The University of Queensland, Brisbane, QLD, Australia; 8 Faculty of Health Studies, University of Sarajevo, Sarajevo, Bosnia and Herzegovina; 9 Department of Bioethics, Faculty of Medicine, University of Otago, Dunedin, New Zealand; 10 Fundação Dom Cabral, (FDC), Nova Lima, MG, Brazil; 11 Unit of Psychiatry, Department of Public Health and Medicinal Administration, Institute of Translational Medicine, Faculty of Health Sciences, Centre for Cognitive and Brain Sciences Institute of Advanced Studies in Humanities and Social Sciences, Taipa, Macao SAR, China

**Keywords:** birth rates, global health, women-centered policymaking, women’s health, women’s rights

## Abstract

Global birth rates have been in steady decline and are projected to continue this trajectory in the coming decades. While existing literature provides important insights into the demographic and socioeconomic dimensions of this trend, there remains a critical gap in theoretical frameworks that engage with the broader implications of declining fertility. Current family planning programs often concentrate on pregnancy and postnatal care but tend to overlook the preconception period, particularly the need to equip women with the resources and autonomy required to make informed decisions about reproduction. Such omissions may have unintended consequences for women’s reproductive choices and broader fertility patterns. Meanwhile, rather than centering policy efforts solely on increasing birth rates, it is imperative to shift the focus toward improving the quality of births which emphasizes the long-term comprehensive benefits to individuals, families and society. This approach necessitates the provision of comprehensive support covering the entire reproductive cycle for women, supported by robust engagement from the global health community. This study seeks to explore the multifaceted factors that shape women’s capacity and inclination to bear children under conditions conducive to positive maternal and infant outcomes. It introduces a holistic framework designed to inform the policies and practices of health and governmental institutions, with the aim of promoting women’s overall well-being and effective and sustainable fertility outcomes.

## Introduction

The global birth rates are in sustained decline (1). These signs, as shown in research and beyond (2–5), underscore the scale and scope of the baby busts happening across the world. Birth or fertility rates are the average number of children a mother gives birth to. Analyses of the population trends paint a sobering picture—23 countries worldwide, including Japan, Spain, Italy, and Ukraine, will see their populations halve by 2,100 (6). The coronavirus disease 2019 (COVID-19) further complicated the situation. Evidence shows that for the United States (U. S.), that despite a slight increase in 2021 compared to the previous year, total births dropped in 2023 by 152,000 per year compared to 2019 (7). Similar trends can also be seen in European and Asian countries (8, 9). China, for instance, witnessed a 15% drop in its number of newborn babies in 2020 compared with that of 2019 (10). In a recently published *Lancet* study, the researchers found that between 2016 and 2021 alone, global annual livebirths have plummeted from 142 million to 129 million (11). The study further suggested that over half (53.9%) of the 204 countries and regions studied will see their birth rates drop below the replacement level (11). Importantly, a decline in the period total fertility rate (TFR) is not inherently negative. In many low- and middle-income countries, particularly where unmet need for contraception and unintended pregnancy remain high, it may partly reflect improved access to contraception and family planning services, enabling women and couples to better realize their fertility intentions and reproductive autonomy regarding whether, when, and how many children to have (12, 13). Only when fertility remains persistently below replacement level does it accelerate population aging, increase the old-age dependency ratio, and generate fiscal and economic pressures (14).

These numbers, overall, paint the contours of one consequential reality that society must confront—the makeup of humanity has experienced seismic changes and will probably have to embrace even greater challenges in the coming decades, if not centuries, that may result in significant “economic, social, environmental, and geopolitical consequences” (6). Declining fertility and shrinking family size also have progressively weakened family-based systems of economic support, caregiving, and emotional risk-sharing, thereby increasing older adults’ vulnerability to a cumulative risk pathway linking social isolation and loneliness to functional decline, disability, and poverty, ultimately exacerbating the societal and health burdens of population aging (15, 16). Among all the potential changes declining birth rates might cause, the unsustainability of the workforce might worry society the most—not only the world population is seeing a sustained and significant birth drop, it is also witnessing a surge in the aging population, which means that the urgency for being equipped with enough labor to address the needs of older adults as well as children might be increasingly pronounced and pressing (17–19). These insights combined underscore the urgent need for the world to design and develop solutions that could effectively generate sufficient productivity to address the rising caregiving needs and other health services.

As with many issues the world faces, the involvement of women as key stakeholders is core to addressing society-wide problems (20–23). To avoid being inundated by the sea of unfamiliar or unprecedented challenges a diminishing population could induce, government officials have been devising ways to prompt women to have more births across societies (24–26). Noticeably, in 2019, the Prime Minister of Hungary encouraged women in his country to have four or more babies, as a way to boost its birth rates (24). China also tries to persuade women to have more babies by replacing the “one-child policy” with a two-child policy, and subsequently, a three-child policy, allowing most couples to be able to have three children instead of one (27, 28). As population data usually take years to solidify and analyse, it will be difficult to know how the Hungarian leader’s 2019 encouragement, along with its other family planning policies, might impact the country’s birth rates in the long run. What is clear, though, is that, so far, Chinese couples’ responses to the two-child policy have been measured and lukewarm, as evidenced earlier, a 15% drop in birth rates has been recorded in China (10).

One way to explain why a large portion of family planning policies worldwide have failed nation after nation is the lack of structured and systematic understanding of what society really needs in light of the growing importance of science and technology and the increasingly enlarging threats of climate change (29–31), whether policies should prioritize more births or higher-quality births. Furthermore, there is also a lack of theoretical underpinning that could help health and government officials understand and address issues rooted in not only women’s abilities, but also their intention, to have births and raise children. This lack of theoretical understanding could hinder policymakers’ abilities to design and develop effective and efficient policies and social systems that could yield meaningful outcomes in terms of an increase in women’s health and quality of life as well as quality birth rates. Thus, to address the research gap, this study attempts to examine drivers that shape women’s abilities and intentions to have quality births. Furthermore, a framework that can help health and government officials to boost quality births, but also improve women’s health and well-being is presented and discussed.

## Limitations of outcome-centered family planning policies

Overall, a wide range of family planning policies introduced by countries across the world to tackle the low birth issue has yielded suboptimal results (32–35). Take Russia for instance, a country that has long been plagued by low birth rates. In the span of decades, the Russian government has adopted a long list of family planning policies, with the “cash-for-babies” policies being the most prominent ones— direct cash payments are being utilized to incentivize couples to have more births (32). Unfortunately, policy after policy, Russia’s birth rates remain less than ideal. In other words, most of Russia’s family planning policies, almost all of which focus on outcome-centered behavioral dynamics, failed to produce desirable results (36).

Similar trends can also be seen in other countries, such as South Korea. Similar to Russia, South Korea also has one of the lowest birth rates globally—in 2020, national, South Korea’s number of births per woman was 0.84, in Seoul, the capital city, the rate drops to 0.64; the situation is expected to become even more sobering—the United Nations estimated that, by 2050, South Korea is going to embrace so few new births that its share of older adults is projected to become the largest worldwide (37). To improve birth rates, South Korea has invested substantial resources to develop effective family planning policies that could boost birth rates, ranging from cash rewards for births, stipends for child-rearing, free nurseries, subsidized pay amid child-care leave, housing bonuses, and support for *in vitro* fertilization, as well as more creative ones such as arranging blind dates for public servants in the hope that these dates can translate into marriages and subsequently, babies (38, 39). However, though already 130 million U. S. dollars have been invested in these policies, there have been little improvements in South Korea’s birth rates.

Given that fertility decisions are jointly negotiated within couples (40), many policy amendments have been aimed at fathers’ earlier involvement in child-rearing and at changing the gendered division of unpaid work and care toward a gender-equal sharing of family responsibilities in general (41), but men’s caregiving involvement remains limited (42).

Similar trends can be seen across nations worldwide that countries face varying degrees of disappointing results from family policy interventions that aim to boost fertility (43, 44). Although family planning policies in these countries have generated measurable short-term fertility gains and alleviated childcare-related financial pressures for some families (45, 46), they have also, in many national contexts, tended to produce a “support in form but pressure in substance” effect in the short run: women may gain access to leave entitlements and financial subsidies, yet simultaneously face heightened risks of employer discrimination and a further entrenchment of gendered caregiving responsibilities (47, 48).

**Figure 1:**
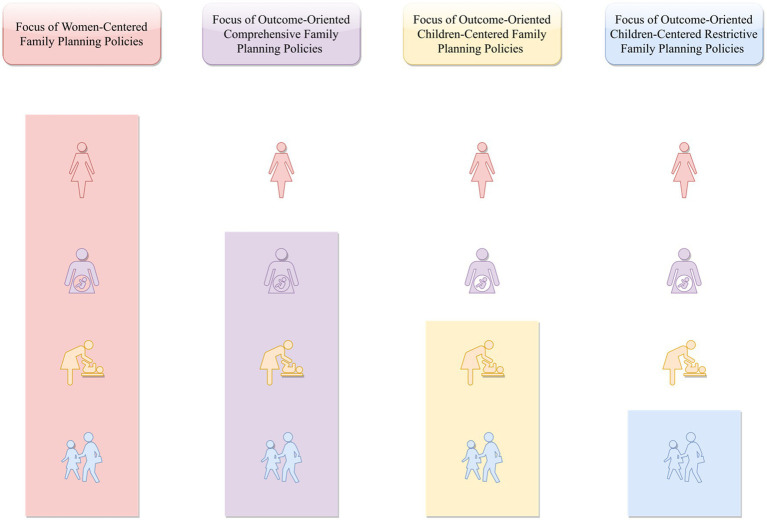
The central focus of women-centered family planning policies compared to conventional outcome-oriented policies that are children-centered.

A growing body of literature suggests that most existing family planning policies remain predominantly outcome-focused (32–35). Specifically, policy support is largely structured around children who are not yet born, while comparatively little attention is paid to the economic, health, and psychosocial conditions that shape women’s readiness to have and raise children (please see Figure 1).

In other words, rather than protecting women’s economic prospects to reduce the financial risks associated with childbearing, strengthening their health to ensure physical readiness, or fostering supportive family and societal environments to enhance psychological and social preparedness, current family planning policies continue to prioritize post-birth outcomes. This outcome-centered orientation may inadvertently sustain high perceived costs of childbearing for women, thereby constraining fertility intentions and limiting the long-term effectiveness of such policies. These structural inadequacies may help explain why many family planning initiatives fail to produce sustained or desirable demographic outcomes.

## Women-centered family policies and reproductive autonomy

Childbirth is not a discrete, one-time event; rather, it is a long-term process embedded within women’s life course. Decisions and practices related to childbirth are shaped not only by women themselves but also by the influence of families, communities, and broader social structures. Throughout this process, women bear the primary physiological risks and social consequences, making the availability of support and resources a decisive factor in shaping both their childbirth experiences and outcomes. Support could include the degree to which women’s health and well-being are being empathetically acknowledged and addressed (e.g., proposal of women-centered healthcare services), whereas resources could be understood as the scale and scope of services, financial subsidies, and other assistance designed and devoted to ensuring support for women is materialized.

Women-centered family planning policies could be viewed as tailored mandates developed to address women’s family planning needs and preferences. Accordingly, women’s reproductive autonomy is defined not merely as reproductive intention, but as the convergence of women’s abilities and intentions to have births and raise children, that is women’s ability to exercise their reproductive choices under enabling social, economic, and institutional conditions. Consequently, women’s reproductive autonomy is closely intertwined with their well-being.

While women’s well-being can be influenced by a myriad of facilitators and barriers to varying degrees, four defining factors dwarf the rest of the drivers, including women’s financial capabilities, physical readiness, available social support, and their psychological readiness. These defining factors are essentially a hierarchy of needs and can be better understood in light of Maslow’s model which ranks motivators of human actions into five levels, from most basic to most advanced: physiological needs (e.g., air, water, food, etc.), safety needs (e.g., safety, security, resources, health, etc.), love and belonging (e.g., family, friendship, intimacy, etc.), esteem (e.g., respect, freedom, status, etc.), and self-actualization (e.g., achieving one’s full potential) (49). The model has been widely utilized, including research on women’s health and well-being (50–56), such as family planning (51–54). Empirical studies show that interventions aimed at improving women’s well-being are effective only when women’s basic needs—such as health, financial security, and social support—are adequately addressed (50, 52). However, existing applications of this framework in family planning research have predominantly focused on mothers, rather than women more broadly across the life course (51–54).

**Figure 2:**
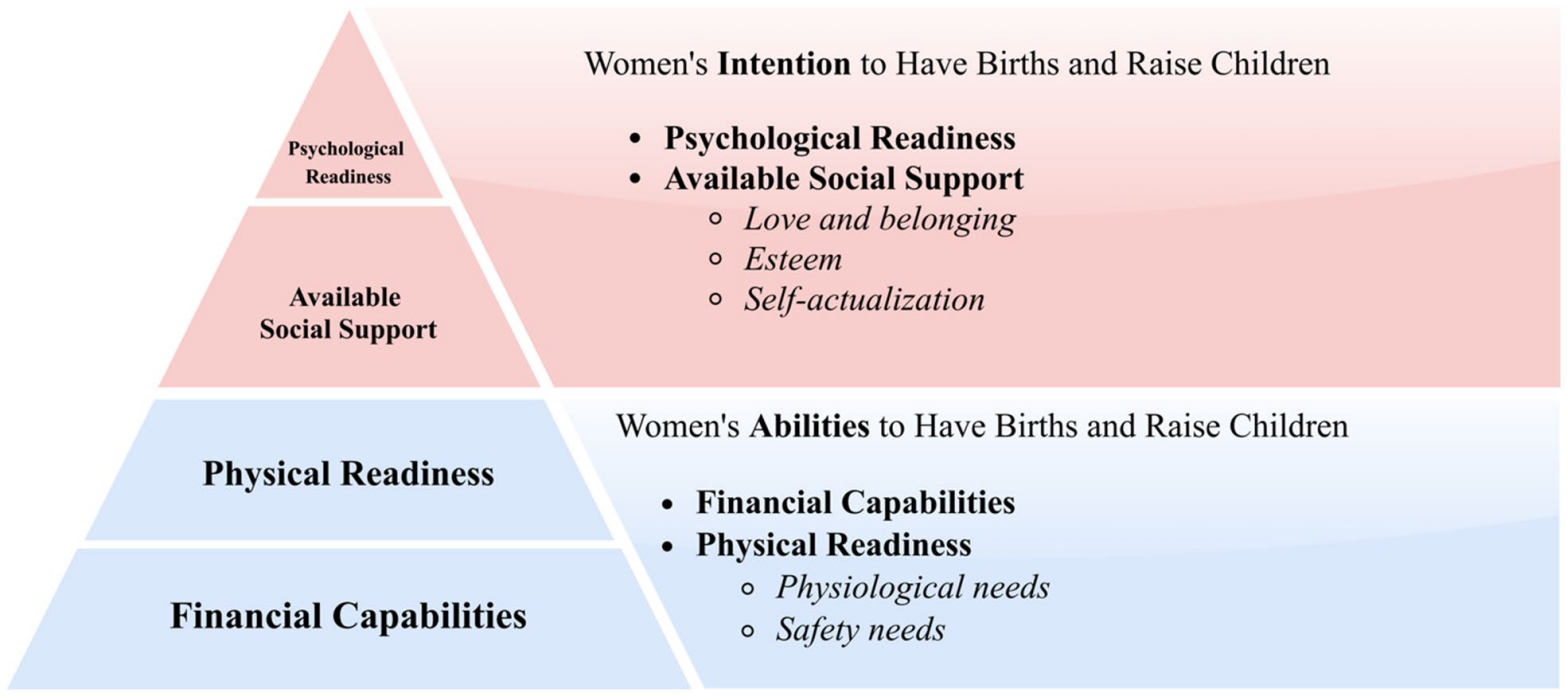
A schematic representation of the defining factors that shape women’s fertility decision.

This study extends Maslow’s hierarchy of needs model in the context of women-centered policies to shed light on the defining factors that shape women’s autonomous fertility decisions. Drawing insights from the model (52), as well as relevant insights from the broader literature, this study further categorizes factors that shape women’s autonomous fertility decisions into two hierarchies: women’s abilities (i.e., financial capabilities and physical readiness) and intention (i.e., available social support and psychological readiness) to have births and raise children. A schematic representation of these factors can be found in Figure 2.

**Figure 3:**
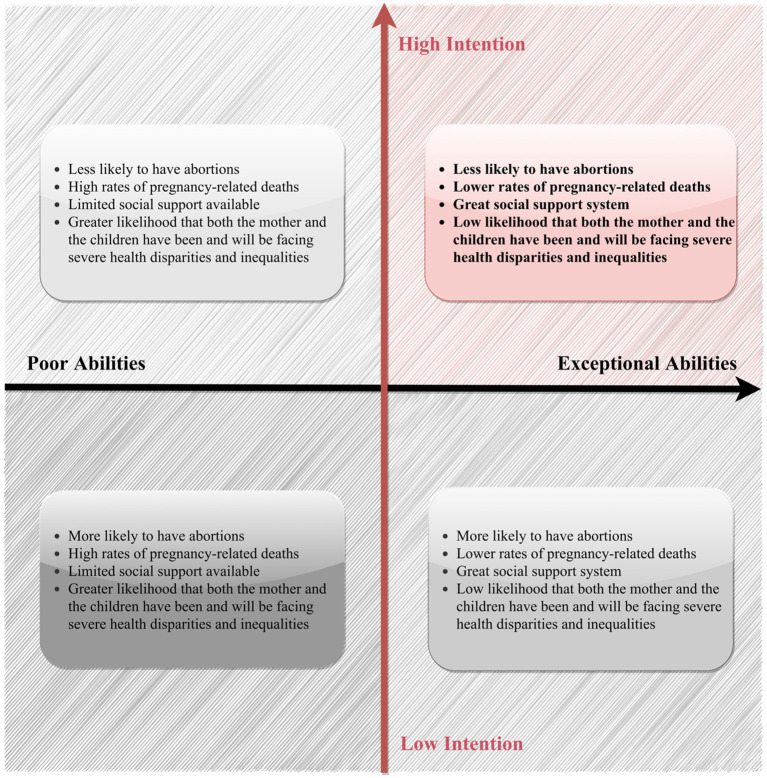
The interplay between women’s abilities (i.e., financial capabilities and physical readiness) and intention (i.e., psychological readiness and available social support) to have births and raise children.

Overall, the proposed framework (Figure 2) can further help explain why outcome-oriented family policies have often proven ineffective. Rather than acknowledging the fact that a large portion of women in society has yet to fulfill their survival-dependent needs (physiological and safety needs), these policies try to persuade women to give up their self-preservation instincts for food, shelter, safety, security, resources, health, etc., in order to have babies that they may also not be able to afford. Under such conditions, women’s material and physical capacities to sustain childbearing and childrearing remain constrained, which in turn undermines the formation and persistence of reproductive intentions. Building on this logic, a detailed illustration of how the interplay of women’s abilities and intention to give birth and raise children influence their autonomous fertility decisions is presented in Figure 3.

## From quantity to quality: rethinking fertility policy priorities

What is “quality birth”? Quality birth is defined as a woman-centered, health- and well-being–oriented concept that spans the entire continuum of reproduction. Specifically, quality birth extends beyond favorable neonatal health outcomes at the time of delivery. It emphasizes women’s ability to attain adequate and sustained protection across physical health, psychological well-being, economic security, and social support before childbirth, during labor and delivery, and throughout the postpartum period. By ensuring such comprehensive support, quality birth seeks to reduce the risks and costs associated with childbearing while generating long-term benefits for individuals, families, and society as a whole. This definition not only aligns with the World Health Organization’s standards for improving the quality of maternal and newborn care, but also explicitly foregrounds women’s autonomy and multidimensional well-being across the full reproductive continuum (57).

A central challenge confronting family planning policy lies in determining whether fertility quantity or fertility “quality” should be prioritized under conditions of resource constraint. Existing quantity-oriented policies have primarily focused on mitigating macro-level risks associated with declining fertility rates and population contraction. However, such approaches have, to some extent, overlooked children’s health and developmental outcomes as well as the long-term social costs embedded in the childbearing process itself.

Under conditions of limited public resources, this dilemma becomes particularly salient. As societies worldwide confront existential challenges such as climate change and resource scarcity, the question of how to allocate finite reproductive-related resources more efficiently has become unavoidable in the design of fertility policies. Notably, children who are born in poor health or in highly vulnerable circumstances not only impose substantial caregiving burdens on families but also generate increased long-term societal costs in health care, education, and social protection systems.

One of the key theoretical frameworks for understanding this issue is the quality–quantity trade-off theory (58). This theory posits that under conditions of constrained household resources, an increase in the number of children necessarily reduces the average investment available to each child. When the returns to child “quality” rise, such as when investments in education and health yield higher human capital returns, households tend to optimize resource allocation by reducing fertility and increasing per-child investments in quality. Moreover, in periods of rapid technological advancement, individuals are required to acquire new skills, and opportunities for personal development expand accordingly. Education and rational decision-making thus become increasingly important. As a result, the costs of childrearing continue to rise, while children’s direct economic contributions to the household steadily decline. At the same time, declines in mortality driven by advances in medical technology have expanded the number of dependents within families, while simultaneously reducing incentives for high fertility. In addition, women have increasingly moved beyond traditional domestic roles to assume new economic and social positions, many of which are progressively less compatible with the demands of childbearing and intensive childrearing (59).

In recent years, the classic quality–quantity trade-off framework has expanded beyond a narrow focus on investments in children’s human capital (such as education and health). Together with mechanisms such as the opportunity cost of maternal time, it has been increasingly applied to explain fertility decision-making and persistently low fertility, highlighting the substantial time burdens and social opportunity costs borne by women in childbearing and childrearing (60). Concurrently, within the field of maternal and child health, authoritative quality frameworks have explicitly incorporated the quality of the childbirth and delivery experience as a core dimension of quality. These frameworks emphasize women’s health, dignity, and access to supportive care, thereby providing both normative and practical foundations for a shift in fertility policy from a quantity-oriented approach toward a quality-oriented paradigm that simultaneously values women’s well-being and children’s development (57). From this expanded perspective, policies that focus exclusively on increasing fertility numbers risk overlooking the long-term social efficiency implications of women’s well-being and the quality of childbirth experiences, potentially undermining the sustainability of reproductive behavior. An extended quality–quantity trade-off framework therefore strengthens the case for institutional investments in women’s health and child development, contributing to the construction of a more sustainable and equitable fertility policy system.

## Reconstructing fertility policy with women’s well-being at the core

**Figure 4:**
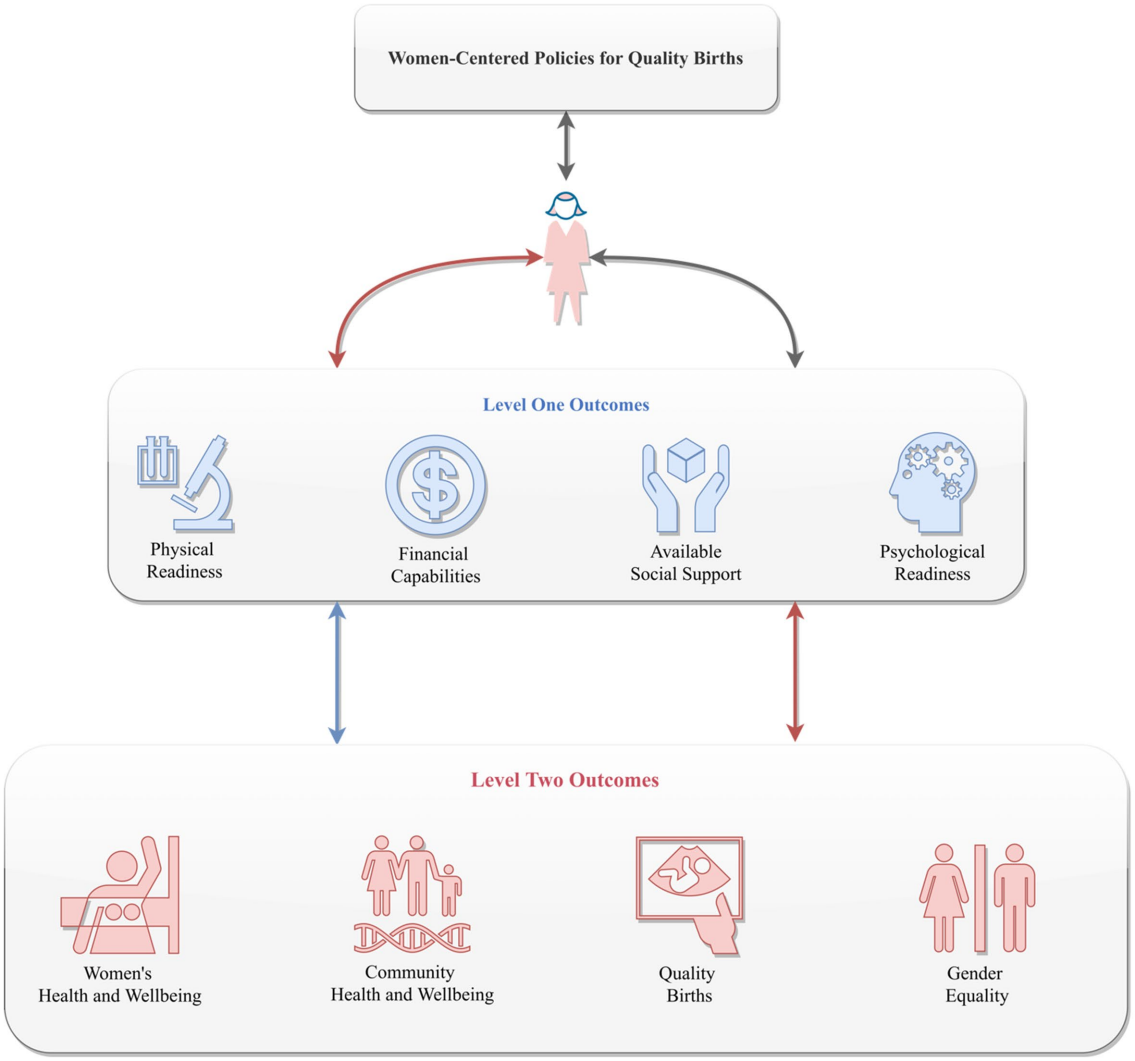
A framework for understanding and developing women-centered policies for quality birth.

Since the 1994 International Conference on Population and Development (ICPD), global population policy has shifted from fertility targets toward reproductive autonomy, rights, and well-being (61). Yet this paradigm remains insufficiently realized in practice. The UNFPA *State of World Population 2025* report shows that women worldwide are systematically unable to achieve their desired fertility, either exceeding or falling short, due to persistent socioeconomic, cultural, and gender-based constraints (62). These insights combined underscore the need for and the importance of women-centered policies for quality births, as well as the potential of the framework to address persistent yet pertinent challenges women face on a daily basis. Overall, putting women’s health and well-being first, rather than those of the babies or the family, could be the most noteworthy advantage of the women-centered policies for quality births, for it has greater potential to generate more sustainable social goods to countries across the world: it delivers support and resources to women that can help them address their most basic and fundamental needs in a timely manner, which in turn, could help alleviate the health and economic challenges women face and make them more empowered, and subsequently, and can help women build their non-survival related needs such as love and belonging, esteem, and self-actualization, including facilitate their intention to have quality births. Therefore, placing women’s well-being at the center of policy design is not only a fundamental requirement of gender equity but also a critical precondition for enhancing the effectiveness and social acceptability of fertility policies. A framework that could help health and government officials to better understand and develop women-centered policies in the context of improving society’s quality births can be found in Figure 4.

Incentive-based policies that rely primarily on financial subsidies tend to alleviate the costs of childbearing only in the short term and are insufficient to alter the structural constraints women face in fertility decision-making. By contrast, *women-centered quality births* emphasize enhancing women’s sense of control over reproductive risks and life-course consequences through strengthened reproductive health services, improved maternal health protection across the perinatal period, greater employment stability, and more robust caregiving support. This shift redirects policy attention away from merely stimulating fertility behavior toward improving the conditions under which childbearing occurs, thereby helping to sustain both fertility intentions and the capacity to realize them over the long term.

## Conclusion

Demography is often described as destiny. In this paper, we argue that long-term demographic trajectories are shaped less by demography per se than by women’s health and well-being, which constitute a fundamental foundation for sustainable population development. Women’s health, security, and quality of life underpin their autonomous fertility intentions, thereby influencing long-term fertility outcomes. Policies that prioritize short-term demographic goals while neglecting the structural conditions necessary for women’s well-being risk undermining both reproductive choice and population sustainability. Safeguarding women’s health and well-being should therefore be recognized not only as a matter of individual rights, but also as a prerequisite for effective and sustainable fertility policy. A long-term, health-oriented, and women-centered policy perspective is therefore essential for addressing contemporary demographic challenges, such as the conceptual framework advanced in this paper. Its policy effects therefore remain to be tested and validated through comparative empirical studies across different welfare-state regimes, levels of health-system capacity, and contexts of gender inequality. Future research should assess the key implementation conditions, potential distributional consequences, and cost-effectiveness of women-centered, health-oriented policy packages in strengthening reproductive autonomy and supporting the realization of completed fertility, thereby generating more actionable evidence to inform sustainable and effective fertility-policy design.

## Data Availability

The original contributions presented in the study are included in the article/supplementary material, further inquiries can be directed to the corresponding author/s.
